# Antagonizing Effects of *Clematis apiifolia* DC. Extract against Benzo[a]pyrene-Induced Damage to Human Keratinocytes

**DOI:** 10.1155/2019/2386163

**Published:** 2019-11-05

**Authors:** Seung Eun Lee, See-Hyoung Park, Ju Ah Yoo, Kitae Kwon, Ji Woong Kim, Sae Woong Oh, Se Jung Park, Jangsoon Kim, Eunbi Yu, Byung Seok Han, Jae Youl Cho, Jongsung Lee

**Affiliations:** ^1^Molecular Dermatology Laboratory and Biocosmetics Research Center, Department of Integrative Biotechnology, College of Biotechnology and Bioengineering, Sungkyunkwan University, Suwon City 16419, Gyunggi Do, Republic of Korea; ^2^Department of Bio and Chemical Engineering, Hongik University, 30016 Sejong City, Republic of Korea; ^3^AMI Cosmetic Co., Ltd., 19 Yanghwa-ro, Mapo-gu, 04026 Seoul, Republic of Korea; ^4^Molecular Immunology Laboratory, Department of Integrative Biotechnology, College of Biotechnology and Bioengineering, Sungkyunkwan University, Suwon City 16419, Gyunggi Do, Republic of Korea

## Abstract

*Background*. Benzo[a]pyrene (B[a]P), a polycyclic aromatic hydrocarbon present in the atmosphere, has cytotoxic and carcinogenic effects. There have been no reports to demonstrate involvement of *Clematis apiifolia* DC. extract (CAE) in B[a]P-induced effects. This study was conducted to investigate the effect of CAE on B[a]P-induced effects and to elucidate its mechanism of action in HaCaT human keratinocytes. CAE inhibited aryl hydrocarbon receptor (AhR) signaling by decreasing both XRE reporter activity and expression of cytochrome P450 1A1 (CYP1A1) induced by B[a]P treatment in HaCaT cells. We also found that B[a]P-induced nuclear translocation of AhR and production of reactive oxygen species (ROS) and proinflammatory cytokines were attenuated by CAE treatment. CAE treatment suppressed B[a]P-induced phosphorylation of Src (Tyr416). In addition, dasatinib, a Src inhibitor, also inhibited B[a]P-induced nuclear translocation of AhR, similar to CAE treatment. In addition, CAE activated antioxidant response element (ARE) signaling by increasing ARE luciferase reporter activity and expression of ARE-dependent genes such as nuclear factor (erythroid-derived 2)-like 2 (Nrf2), NAD(P)H dehydrogenase [quinone] 1 (NQO1), and heme oxygenase-1 (HO-1). Nuclear translocation of Nrf2 by CAE was demonstrated by Western blot analysis and immunocytochemistry. The effects of CAE on ARE signaling were attenuated by knockdown of the Nrf2 gene. Inhibition of AhR signaling and activation of antioxidant activity by CAE operated in a reciprocally independent manner as evidenced by AhR and Nrf2 siRNA experiments. These findings indicate that CAE exerts protective effects against B[a]P by inhibiting AhR signaling and activating Nrf2-mediated signaling, suggesting its potential in protection from harmful B[a]P-containing pollutants.

## 1. Introduction

Since human skin covers the outer surface of the body, it is always exposed to various stressors [1, 2]. In particular, epidermal keratinocytes are very susceptible to the oxidative stress caused by environmental pollutants, which induce cancer, aging, inflammatory disorders, and vitiligo in the skin [3, 4]. Reactive oxygen species (ROS), including most free radicals, can damage cellular proteins, lipids, and DNA [5]. Therefore, approaches for protecting skin against oxidative stress are important and required.

Benzo[a]pyrene (B[a]P), a main environmental pollutant, is a type of polycyclic aromatic hydrocarbon with cytotoxic and carcinogenic effects [6]. B[a]P induces generation of ROS, which is mediated by activation of aryl hydrocarbon receptor (AhR) [7]. AhR is abundantly expressed in the epidermal keratinocytes and plays a role as a xenobiotic chemical sensor [8]. Upon activation with its ligands, AhR moves from the cytoplasm into the nucleus, where it upregulates the transcription of target genes by binding to xenobiotic-responsive element (XRE), its specific DNA recognition sequence in the promoters of target genes [9]. Activation of AhR induces expression of its target gene cytochrome P450 1A1 (CYP1A1) [10]. The enhanced expression of CYP1A1 induces ROS production, leading to protein and DNA damage [11]. At present, research on materials that can inhibit and attenuate the B[a]P effect is continuously being carried out. Several natural products have been reported to attenuate the B[a]P effects such as *Morus alba* L. extract [12] and cinnamaldehyde [13].

To maintain normal skin biology, overproduced ROS should be reduced to normal levels by endogenous antioxidant enzymes including NAD(P)H dehydrogenase [quinone] 1 (NQO1) and heme oxygenase-1 (HO-1) [14]. Nuclear factor (erythroid-derived 2)-like 2 (Nrf2) plays a master role in antioxidant signaling by inducing expression of antioxidant enzymes [15]. Specifically, under physiological conditions, Nrf2 remains in the cytoplasm in the form of the Nrf2-Keap1-CUL3 complex [16]. However, under oxidative conditions, Nrf2 translocates to the nucleus by dissociating from Keap1 and induces transcription of antioxidant genes [17].

*Clematis apiifolia* DC. is native to South Korea, Japan, and China. According to *Donguibogam*, a Korean traditional medicine encyclopedia, *Clematis apiifolia* DC. improves neuralgia, facial nerve paralysis, muscle paralysis, rheumatoid arthritis, and more. However, there have been no scientific reports on the effects of the extract on human physiology, especially on skin biology. Therefore, in this study, we investigated the effects of *C. apiifolia* DC. extract (CAE) on B[a]P-induced damage to HaCaT cells and its mechanisms of action.

## 2. Materials and Methods

### 2.1. Cell Culture and Reagents

HaCaT cells (American Type Culture Collections, Manassas, VA, USA), a human keratinocyte cell line, were cultured and maintained in Dulbecco's modified Eagle's medium (DMEM, Thermo Fisher Scientific, Inc., Waltham, MA, USA) supplemented with 1% antibiotics (penicillin/streptomycin) and fetal bovine serum (FBS, 10%) at 37°C in a 5% CO_2_ humidified incubator. The HEK293-TRPV1-luciferase stable cell line (Creative Biogene Biotechnology, Shirley, NY, USA) was cultured in DMEM supplemented with 10% FBS, 10% puromycin, and 1% antibiotics at 37°C in a 5% CO_2_ humidified incubator. The following are the cell culture reagents: B[a]P (CAS No. 50-32-8, purity 99.9%, Sigma-Aldrich Co., N.Y., USA), dasatinib (Src inhibitor, Sigma-Aldrich Co.), AhR antibodies (Santa Cruz Biotechnology, Santa Cruz, Ca, USA), ARNT antibodies (Santa Cruz Biotechnology), LaminB1 antibodies (Epitomic, Burlingame, CA, USA), *α*-tubulin antibodies (Epitomic), *β*-actin antibodies (Sigma-Aldrich Co.), phospho-Src (Tyr416) antibodies (Cell Signaling Technology Inc., Beverly, MA, USA), Src antibodies (Cell Signaling Technology Inc.), CYP1A1 antibodies (Santa Cruz Biotechnology), NQO1 antibodies (Santa Cruz Biotechnology), and HO-1 antibodies (Santa Cruz Biotechnology).

### 2.2. Plant Material, Extraction, and Determination of Total Phenols and Flavonoids

*Clematis apiifolia* DC. samples were collected from Jeju Island (Republic of Korea) in 2018. Taxonomic identification was conducted by a botanist at Ami Cosmetic Inc. (Republic of Korea). A voucher specimen was deposited in the Ami Cosmetic Inc. Research Center. *Clematis apiifolia* DC. samples were washed with water and then dried under shade and ventilation. The dried bark was ground using an electronic miller. The powder was extracted using 70% ethanol for 72 h at room temperature, filtered through Whatman filter paper No. 1, and concentrated using a rotary evaporator. The concentrated retentate was aliquoted and stored at −70°C. The content of total phenols was measured by a spectrophotometer, using gallic acid as a standard, according to the method described by the International Organization for Standardization (ISO) 14502-1. In brief, an aliquot of diluted sample extract (1.0 ml) was transferred in duplicate to separate tubes containing a 1/10 dilution of Folin-Ciocalteu's reagent in water (5.0 ml), and a sodium carbonate solution (4.9 ml, 7.5% *w*/*v*) was added. The tubes were then incubated at room temperature for 60 min before absorbance at 765 nm was measured against water. The content of total phenols was expressed as gallic acid equivalents in g/100 g extract. The concentration of polyphenols in samples was derived from a standard curve of gallic acid. In addition, in order to determine total flavonoids, distilled water (1 ml) was added to the samples (0.25 ml of the extracts). 5% NaNO_2_ (0.075 ml), 10% AlCl_3_ (0.075 ml), and 1 M NaOH (0.5 ml) were then added sequentially at 0.5 and 6 min. Finally, the volume of the reacting solution was adjusted to 2.5 ml with double-distilled water. The absorbance of the solution at a wavelength of 410 nm was detected using an Epoch spectrophotometer. Quercetin, a ubiquitous flavonoid present in many natural extracts, was used as a standard to quantify the total flavonoid content. Results were expressed in microgram quercetin equivalents/100 g extract.

### 2.3. DCFDA-Cellular Reactive Oxygen Species (ROS) Detection Assay

The ROS levels were measured by a DCFDA-cellular reactive oxygen species detection assay kit (ab113851, Abcam, Cambridge, UK) using a fluorescence microscope and microplate. Cells were seeded and then incubated with CAE or *tert*-butyl hydroperoxide (TBHP) solution as a positive control. After 24 h, cells were washed twice in PBS and stained with DCFDA (25 *μ*M) in PBS for 15 min at 37°C under dark conditions. Stained cells were washed, and their signals were detected at Ex/Em: 485/535 nm. This determined the change as a percentage of the control after background subtraction.

### 2.4. Small-Interference RNA (siRNA) for Nrf2 and AhR

The ON-TARGETplus SMARTpool human siRNAs against Nrf2 (L-004018-00-0020), AhR (L-004990-00-0020), and ON-TARGETplus nontargeting siRNA (D-001810-10-05) were provided by Thermo Fisher Scientific, Inc. (Waltham, MA, USA). Cells were transfected with the indicated siRNAs at 50 nM for 24 h using the DharmaFECT transfection agent (Dharmacon Research, Lafayette, CO, USA), according to the manufacturer's protocols.

### 2.5. Analysis of mRNA Levels Using Real-Time RT-PCR

Real-time RT-PCR analysis was performed using an ABI7900HT Instrument (Applied Biosystems, Waltham, MA, USA). For TaqMan analysis, predesigned or optimized assays on demand (Applied Biosystems) were used, including Nrf2 (ID: Hs00975961_g1), NQO1 (ID: Hs01045993_g1), AhR (ID: Hs00169233_m1), CYP1A1 (ID: Hs01054796_g1), glyceraldehyde-3-phosphate dehydrogenase (GAPDH) (ID: Hs00266705_g1), hypoxanthine-guanine phosphoribosyltransferase (HPRT) (Hs02800695_m1), and 18S (Hs03003631_g1). The data were analyzed using ABI Sequence Detector Software version 2.0 (Applied Biosystems). Total RNA was extracted from cells using TRI reagent® according to the manufacturer's instructions and stored at -70°C until use. cDNA was synthesized from total RNA (1 *μ*g) using MuLV reverse transcriptase according to the manufacturer's instructions. Real-time RT-PCR analysis was conducted as previously described [18]. The results were normalized to the expression level of endogenous GAPDH and were also tested against two additional housekeeping genes (18S and HPRT). We found that the results were not significantly different from those obtained using GAPDH. Expression levels of target genes were normalized to the levels observed in controls. Results were verified through four-time repetition of the same experiment, each of which was conducted in triplicate.

### 2.6. Enzyme-Linked Immunosorbent Assay (ELISA)

The Multi-Analyte Profiler ELISArray Kit (SABiosciences, Frederick, MD, USA) and IL-8 ELISA Kit (Invitrogen, CA, USA) were used to determine levels of IL-1*α*, IL-6, IL-8, and TNF-*α*, according to the manufacturer's protocols. Absorbance was determined using a LabSystems Multiskan MS Analyser (Thermo Bio-Analysis Japan, Tokyo, Japan). The results were confirmed in three independent experiments.

### 2.7. Assay for Luciferase Reporter

Cells were transfected with XRE (Stratagene, La Jolla, CA, USA) or antioxidant response element- (ARE-) Luc reporters (Addgene, MA, USA), along with 1 *μ*g of the Renilla-luciferase expression plasmid (Promega, Madison, WI, USA) (internal standard) using the DharmaFECT® Duo transfection reagent (Thermo Fisher Scientific, Inc.) according to the manufacturer's recommendations. After 24 h, cells were treated and incubated with CAE for 24 h. The cells were then harvested and subjected to luciferase activity using the Dual Luciferase Assay system (Promega) on the LB953 luminometer (Berthold, Germany). The data were expressed as a ratio of the XRE- or ARE-dependent firefly-luciferase activity to the thymidine-kinase Renilla-luciferase activity (% control). The data were verified with three independent experiments.

### 2.8. Western Blot Analysis

Cell lysates were prepared and separated by sodium dodecyl sulfate-polyacrylamide gel electrophoresis. The gels were blotted onto polyvinylidene difluoride membranes and then incubated with primary antibodies (CYP1A1, *β*-actin, Nrf2, AhR, Src, phospho-Src (Tyr416), AhR nuclear translocator (ARNT), or NQO1). The protein bands were detected with horseradish peroxidase- (HRP-) conjugated secondary antibodies using an enhanced chemiluminescence system (Amersham Biosciences, Piscataway, NJ, USA). The data were verified with three independent experiments.

### 2.9. Preparation of the Nuclear and Cytoplasmic Fractions

The nuclear fractions were prepared using NE-PER Nuclear and Cytoplasmic Extraction reagents (78833, Thermo Scientific) according to the manufacturer's recommendations and then subjected to Western blot analysis for target proteins.

### 2.10. Immunocytochemistry

The cells were fixed using 4% paraformaldehyde in PBS for 15 min and permeabilized in 0.1% Triton X-100 and 0.01% Tween-20 for 20 min at room temperature. After blocking the cells with PBS containing 3% bovine serum albumin (BSA), the cells were incubated with anti-Nrf2 (1 : 200; Cell Signaling) antibodies. After being washed three times, they were incubated with Flamma 594 secondary antibodies (BioActs, Seoul, Republic of Korea). The cells were then mounted on glass slides after counterstaining with DAPI and observed under an LSM 700 laser scanning confocal microscope (Zeiss, Jena, Germany) with a C-Apochromat 20x objective. For the measurement of immunofluorescence intensity, images were captured with the same laser power and the mean intensity of fluorescence signals was determined. Data were analyzed using ZEN 2012 Blue (Zeiss) and ImageJ software (National Institutes of Health, Bethesda, MD, USA), under the same processing parameters.

### 2.11. Statistical Analysis

All data are presented as the mean ± SD. One-way analysis of variance (ANOVA) and Tukey's multiple comparison test with GraphPad Prism (5.0) (GraphPad, La Jolla, CA, USA) were introduced to examine the comparison between the control and the sample groups. A *p* value < 0.05 was considered statistically significant.

## 3. Results

### 3.1. CAE Inhibits B[a]P Effects on Xenobiotic Response Element- (XRE-) Mediated Signaling in HaCaT Cells

**Figure 1:**
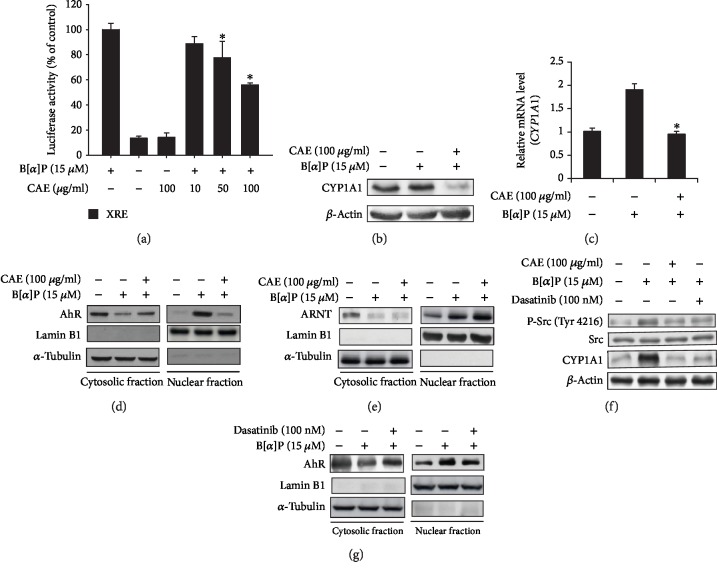
CAE suppresses benzo[a]pyrene (B[a]P) effects on xenobiotic response element- (XRE-) mediated signaling in HaCaT cells. (a) HaCaT cells were transfected with the XRE-Luc reporter and a Renilla-luciferase plasmid using the DharmaFECT® Duo transfection reagent. After 24 h, the cells were treated and incubated with CAE for 14 h in the presence or absence of B[a]P. The cells were then harvested and subjected to a luciferase activity assay. ^∗^*p* < 0.05 vs. B[a]P-treated control. The results were confirmed by repeating three independent experiments. Data are expressed as the mean ± S.D. (b, c) HaCaT cells were incubated with CAE for 24 h in the presence or absence of B[a]P (15 *μ*M) and then subjected to Western blot analysis (b) and real-time PCR analysis (c) for *CYP1A1*. The results were confirmed by repeating the three independent experiments. Data are expressed as the mean ± S.D.^∗^*p* < 0.05 vs. B[a]P-treated control. (d, e, g) HaCaT cells were incubated with CAE (100 *μ*g/ml) or dasatinib in the presence of B[a]P (15 *μ*M) for 24 h and then subjected to Western blot analysis after preparation of the nuclear and cytoplasmic fractions. (f) HaCaT cells were incubated with dasatinib (100 nM) in the presence of B[a]P (15 *μ*M) for 24 h and then subjected to Western blot analysis. CAE: *Clematis apiifolia* DC.

To examine the impact of CAE on B[a]P-induced effects in human keratinocytes, we performed an XRE-luciferase reporter assay, Western blot, and real-time PCR analyses for CYP1A1 in HaCaT cells. As shown in Figure 1(a), CAE suppressed B[a]P-induced activation of the XRE reporter in a concentration-dependent manner. In addition, the expression of *CYP1A1* was affected by CAE treatment. As shown in Figures 1(b) and 1(c), protein and mRNA levels of the *CYP1A1* gene increased in response to B[a]P treatment. However, CAE treatment attenuated the B[a]P effects on the expression of CYP1A1. As shown by the XRE reporter assay, Western blot, and real-time PCR analyses, B[a]P-induced nuclear translocation of AhR was also reduced by CAE treatment (Figure 1(d)). However, CAE treatment showed no effects on B[a]P-induced nuclear translocation of AhR nuclear translocator (ARNT) (Figure 1(e)). To elucidate inhibitory mechanisms of CAE on B[a]P-induced nuclear translocation of AhR, we examined involvement of Src in the CAE effects. As shown in Figure 1(f), while B[a]P induced tyrosine phosphorylation of Src, CAE treatment suppressed the B[a]P effect. In addition, the increased expression of *CYP1A1* by B[a]P was reduced by CAE treatment. Dasatinib, a Src inhibitor, also attenuated the B[a]P effects on the expression of CYP1A1. Furthermore, dasatinib suppressed the B[a]P-induced nuclear translocation of AhR. These results indicate that CAE antagonizes the effects of B[a]P on human keratinocytes and that Src is involved in the CAE effects.

In addition, the total phenol content of CAE was 94.98 ± 16.988 mg GAE/100 g extract, and the total flavonoid content was 37.94 ± 3.60 mg QUE/100 g extract (Table 1).

### 3.2. CAE Reduces Benzo[a]pyrene- (B[a]P-) Induced Production of Reactive Oxygen Species (ROS)

**Figure 2:**
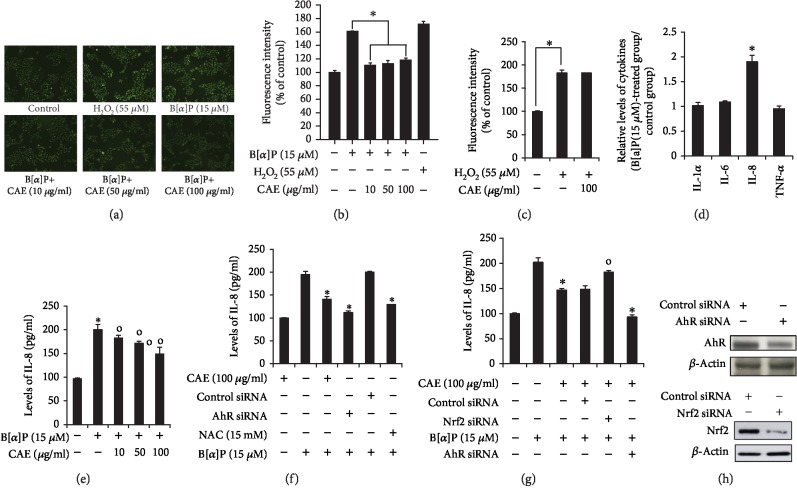
CAE decreases benzo[a]pyrene- (B[a]P-) induced production of reactive oxygen species (ROS) in HaCaT cells. HaCaT cells were incubated with CAE in the presence of B[a]P (15 *μ*M) for 24 h and then subjected to fluorescence image analysis (a) and fluorescence intensity analysis (b). HaCaT cells were incubated with CAE in the presence of H_2_O_2_ (55 *μ*M) for 24 h and then subjected to fluorescence intensity analysis (c). The data were confirmed by repeating three independent experiments. Data are expressed as the mean ± S.D.^∗^*p* < 0.05 vs. B[a]P-treated control. (d, e) HaCaT cells were incubated with CAE in the presence of B[a]P (15 *μ*M) for 24 h and then subjected to an ELISA for proinflammatory cytokines. The results were confirmed by repeating three independent experiments. Data are expressed as the mean ± S.D.^∗^*p* < 0.05 vs. untreated control; ^o^*p* < 0.05 vs. B[a]P-treated control. (f–h) HaCaT cells were transfected with siRNA for the AhR gene or Nrf2 gene using the DharmaFECT® Duo transfection reagent. After 24 h, the cells were incubated with CAE (100 *μ*g/ml) in the presence of B[a]P (15 *μ*M) for 24 h and then were subjected to an ELISA for IL-8 (f, g) and Western blot analysis for AhR or Nrf2 (h). The results were confirmed by repeating three independent experiments. Data are expressed as the mean ± S.D.^∗^*p* < 0.05 vs. B[a]P-treated control; ^o^*p* < 0.05 vs. B[a]P plus CAE-treated control. CAE: *Clematis apiifolia* DC.; NAC: N-acetyl cysteine.

To investigate the involvement of CAE in B[a]P-induced ROS production, we performed a DCFDA-cellular ROS detection assay. As shown in Figure 2(a), while B[a]P increased the levels of ROS, these increased levels were reduced by CAE treatment, as evidenced by ROS imaging (Figure 2(a)) and fluorescence intensity assay (Figure 2(b)). We also found that CAE treatment had no effects on H_2_O_2_-induced ROS production (Figure 2(c)). These data indicate that CAE inhibits the B[a]P effect on ROS production and had no antioxidant activity to directly remove ROS. In addition, we examined effects of CAE on B[a]P-induced production of proinflammatory cytokines. As shown in Figure 2(d), B[a]P induced interleukin-8 (IL-8) production but not that of IL-1*α*, IL-6, or TNF-*α*. The B[a]P effect on IL-8 was reduced by CAE treatment (Figure 2(e)). In addition, knockdown of AhR decreased B[a]P-induced IL-8 production (Figure 2(f)). Treatment with N-acetyl cysteine, an antioxidant and ROS-reducing agent, also reduced the B[a]P-induced IL-8 production (Figure 2(f)). Furthermore, the CAE effect on B[a]P-induced IL-8 production was attenuated by knockdown of Nrf2 (Figure 2(g)). Knockdown of AhR and CAE treatment showed a much stronger inhibitory effect than that of CAE treatment alone (Figure 2(g)). AhR siRNA and Nrf2 siRNA successfully knocked down AhR and Nrf2 proteins in HaCaT cells compared with its level in cells transfected with control siRNA (Figure 2(h)). These data indicate that CAE has antioxidant and anti-inflammatory effects and that the CAE effect on B[a]P-induced IL-8 production is mediated by reduction of ROS levels. In addition, these results indicate that the CAE effect is mediated by both Nrf2 activation and AhR inhibition.

### 3.3. CAE Activates Nrf2-Mediated Signaling

**Figure 3:**
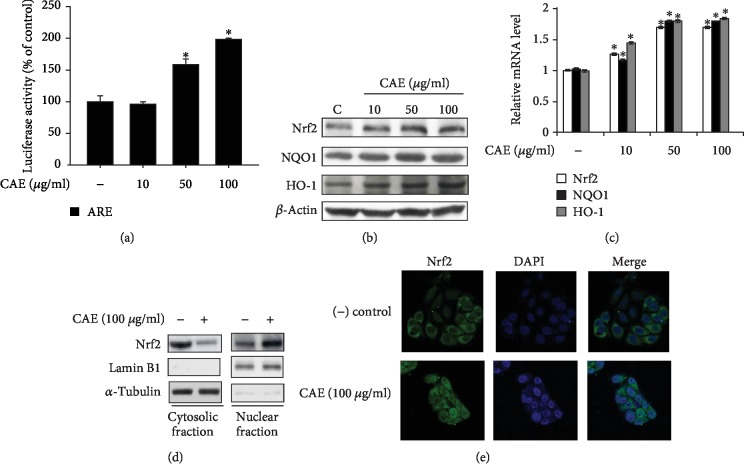
CAE activates Nrf2-mediated signaling. (a) HaCaT cells were transfected with the antioxidant response element- (ARE-) Luc reporter and a Renilla-luciferase expression plasmid using the DharmaFECT® Duo transfection reagent. After 24 h, the cells were incubated with CAE for 14 h. The cells were then harvested and subjected to a luciferase reporter assay. ^∗^*p* < 0.05 vs. untreated control. The results were confirmed by repeating three independent experiments. Data are expressed as the mean ± S.D. (b, c) HaCaT cells were incubated with CAE for 24 h and subjected to Western blot analysis (b), real-time PCR analysis (c) for Nrf2, NQO1, and HO-1, and nuclear translocation analysis for Nrf2 using Western blot analysis (d) and immunocytochemistry (e).

In the previous experiments, we found that CAE contributes to suppression of B[a]P effects in human keratinocytes. We also examined the involvement of CAE in the expression of antioxidant genes. As a first step, an ARE luciferase reporter assay was performed. As shown in Figure 3(a), CAE increased the ARE reporter activity in a concentration-dependent manner. In addition, protein and mRNA levels of the Nrf2 gene were upregulated by CAE treatment (Figures 3(b) and 3(c)). As expected, the expression of NQO1 and HO-1, Nrf2-dependent genes, also increased with CAE treatment (Figures 3(b) and 3(c)). Furthermore, we found that CAE treatment increased nuclear translocation of Nrf2 in both Western blot analysis (Figure 3(d)) and immunocytochemistry assay (Figure 3(e)).

**Figure 4:**
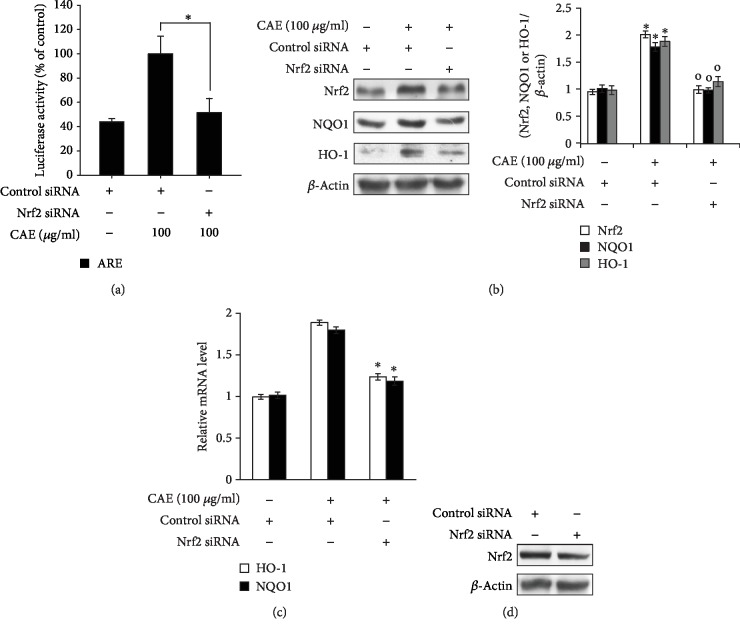
The CAE-induced activation of ARE signaling was attenuated by knockdown of Nrf2. (a) HaCaT cells were transfected with the ARE-Luc reporter and siRNA for the Nrf2 gene using the DharmaFECT® Duo transfection reagent. After 24 h, the cells were incubated with CAE (100 *μ*g/ml) for 14 h and then subjected to the luciferase reporter assay. ^∗^*p* < 0.05 vs. CAE-treated control. The results were confirmed by repeating three independent experiments. Data are expressed as the mean ± S.D. (b, c) HaCaT cells were transfected with siRNA for the Nrf2 gene using the DharmaFECT® Duo transfection reagent. After 24 h, the cells were incubated with CAE (100 *μ*g/ml) for 14 h. The cells were then subjected to Western blot (b, d) and real-time PCR (C) analyses for Nrf2, NQO1, and HO-1. ^∗^*p* < 0.05 vs. untreated control; ^o^*p* < 0.05 vs. CAE-treated control. The results were confirmed by repeating three independent experiments. Data are expressed as the mean ± S.D.

Moreover, as shown in Figure 4, we found that CAE-induced ARE activation was mediated by Nrf2. Specifically, knockdown of Nrf2 using siRNA attenuated the CAE effects such as ARE activation (Figure 4(a)) and upregulation of NQO1 and HO-1 genes (Figures 4(b) and 4(c)). These data indicate that CAE induces ARE-dependent signaling by activating Nrf2.

### 3.4. CAE-Induced Activation of NRF2/HO1 Pathway Was Not Linked with AHR Activation

**Figure 5:**
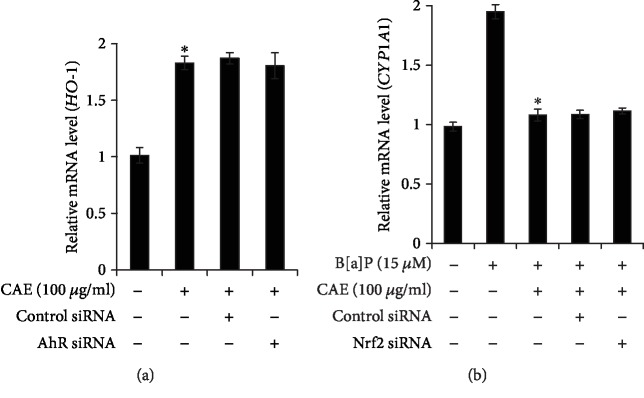
CAE-induced activation of NRF2/HO1 pathway was not linked with AHR activation. (a, b) HaCaT cells were transfected with control siRNA, AhR siRNA, or NrF2 siRNA using the DharmaFECT® Duo transfection reagent. After 24 h, the cells were incubated with CAE (100 *μ*g/ml) in the presence of B[a]P for 24 h and then subjected to real-time PCR analysis for *HO-1* and *CYP1A1*. ^∗^*p* < 0.05 vs. CAE-treated control (a). ^∗^*p* < 0.05 vs. B[a]P-treated control (b). ^o^*p* < 0.05 vs. B[a]P+CAE-treated control. The results were verified by repeating three independent experiments. Data are expressed as the mean ± S.D.

Although some molecules are reported to induce the Nrf2-mediated upregulation of HO-1 via AhR activation [19], CAE did not activate AhR but rather suppressed its function. Therefore, we investigated the AhR dependency of HO-1 induction by CAE using AhR-knockdown keratinocytes transfected with AhR siRNA. As shown in Figure 5(a), CAE-induced HO1 upregulation was not affected in the AhR-knockdown keratinocytes compared with those transfected with control siRNA. In addition, we examined the involvement of the NrF2 pathway in the inhibitory action of CAE on AhR-mediated *CYP1A1* upregulation using Nrf2 siRNA silencing. As shown in Figure 5(b), while CAE significantly inhibited the B[a]P-induced CYP1A1 upregulation, Nrf2 siRNA transfection did not alter the CAE effect. These results indicate that different mechanisms are involved in CAE-induced AhR inhibition and Nrf2 activation.

## 4. Discussion

*Clematis apiifolia* DC. has been traditionally used in Korea as an alternative medicine for various diseases including neuralgia, facial nerve paralysis, muscle paralysis, and rheumatoid arthritis. However, there have been no scientific reports demonstrating its biological activities. In particular, dermatological research has not been conducted on its efficacy. In this study, we investigated involvement of CAE in cytoprotection against B[a]P in human keratinocytes. Similar to protective effect of mulberry (Morus alba L.) extract [12], CAE suppressed B[a]P-induced AhR activation signaling by inhibiting the nuclear translocation of AhR. In addition, CAE activated the Nrf2-mediated signaling pathway. These properties of CAE may contribute to cytoprotection against environmental pollutants.

XRE-dependent signaling is induced in response to external stresses. This signaling is controlled by activation of AhR, a xenobiotic chemical sensor. AhR is a ligand-activated transcription factor that integrates environmental, dietary, microbial, and metabolic cues to control complex transcriptional programs in a ligand-specific, cell-type-specific, and context-specific manner [20]. The AhR signaling pathway has been reported to damage cells and tissues [21]. Specifically, it results in the production of ROS and proinflammatory cytokines [22]. In this study, CAE significantly reduced the B[a]P-induced production of ROS and IL-8. In addition, N-acetyl cysteine, an acetylated variant of the amino acid L-cysteine with antioxidant activity [23], attenuated the production of IL-8 by B[a]P. These data indicate that CAE exerts antioxidant and anti-inflammatory effects and suggest that the antagonizing effects of CAE on B[a]P-induced production of IL-8 are mediated through the CAE-induced reduction of ROS.

IL-8 is one of the major proinflammatory cytokines and is involved in the development of skin diseases such as acne, psoriasis, and palmoplantar pustulosis [24–26]. IL-8 induces these skin diseases by activating the recruitment and function of neutrophils [27]. Propionibacterium acnes is a known inducer of IL-8 production, leading to skin pathologies [28]. Environmental pollutants such as particulate matter (PM) and diesel gas also contribute to the development of skin symptoms [29, 30]. In addition, B[a]P, a polycyclic aromatic hydrocarbon (PAH) in particulate matter, can induce IL-8 production by activating the AhR-ROS pathway [31]. In our study, we found that CAE suppressed B[a]P-induced production of ROS and IL-8. These data suggest that CAE can be used as an antagonistic agent against AhR to improve PM- or environmental stressor-induced symptoms of inflammatory skin diseases.

According to *Donguibogam*, a Korean traditional medicine encyclopedia, although *Clematis apiifolia* DC. has been traditionally used for treatment of neuralgia, facial nerve paralysis, muscle paralysis, and rheumatoid arthritis, scientific demonstrations have not been conducted. In this study, for the first time, we investigated the effects of *C. apiifolia* DC. extract (CAE) on B[a]P-induced damage to keratinocyte biology and its mechanisms of action. In addition, we demonstrated the possibility of CAE as a dermatological approach in the treatment of oxidative stress-induced skin diseases.

Src is a protooncogene tyrosine-protein kinase that belongs to a family of nine nonreceptor tyrosine kinases [32]. Src is located at cell-matrix adhesions and is activated by various signals, including epidermal growth factor (EGF), hepatocyte growth factor (HGF), platelet-derived growth factor (PDGF), vascular endothelial growth factor (VEGF), integrin, and Eph receptor (EphA2) [33, 34]. Some recent reports have demonstrated that Src is involved in the AhR signaling pathway [35]. In our study, CAE treatment suppressed B[a]P-induced tyrosine phosphorylation of Src. In addition, dasatinib, a Src inhibitor, inhibited B[a]P-induced nuclear translocation of AhR and CYP1A1 expression. These data suggest that Src is involved in the CAE effects on nuclear translocation of AhR and expression of its target genes.

Vitiligo is a hypopigmentary skin disorder, and its pathogenesis has not been clearly elucidated. It is clinically characterized by development of white macules due to loss of functioning melanocytes in the skin [36] and believed to be mainly a result of oxidative stress-induced destruction of melanocytes and obstruction of the melanin synthesis pathway [37]. In this study, we found that CAE activated Nrf2 and induced its nuclear translocation, leading to upregulation of the HO-1 and NQO1 antioxidant genes. The CAE-induced activation of the Nrf2/HO-1/NQO1 antioxidant system was found to alleviate the ROS production triggered by B[a]P in keratinocytes. Although most antioxidant phytochemicals upregulate Nrf2 signaling in association with AhR activation [38], similar to action of cinnamaldehyde [13], CAE-induced activation of Nrf2 occurred independently of AhR inhibition, as shown by production of the same effect in both the AhR-knockdown and wild-type keratinocytes. In addition, CAE inhibited AhR activation independently of Nrf2. Although further study is required, these dual effects of CAE are expected to be particularly beneficial in the treatment of vitiligo and other disorders caused by oxidative stress.

## 5. Conclusions

Taken together, these data demonstrate that CAE inhibits AhR activation and upregulates the Nrf2/HO-1/NQO1 antioxidant system. These results suggest that CAE could be used as a possible treatment for vitiligo and other disorders caused by oxidative stress.

## Figures and Tables

**Table 1:** Total phenolic and flavonoid contents in CAE.

Total phenolic contents (mg GAE/g)	Total flavonoid contents (mg QUE/g)
104.451 ± 0.579	43.77 ± 2.69

## Data Availability

The data used to support the findings of this study are available from the corresponding authors upon request.

## Abbreviations

CAE: *Clematis apiifolia* DC. extract
B[a]P: Benzo[a]pyrene
ARE: Antioxidant response element
XRE: Xenobiotic response element
ROS: Reactive oxygen species
AhR: Aryl hydrocarbon receptor
ARNT: AhR nuclear translocator
Nrf2: Nuclear factor (erythroid-derived 2)-like 2
NQO1: NAD(P)H dehydrogenase [quinone] 1
HO-1: Heme oxygenase-1
CYP1A1: Cytochrome P450 1A1.
